# Preparedness for working with genomic medicine among Swedish non-genetics physicians – a nation-wide survey study

**DOI:** 10.1007/s12687-026-00858-y

**Published:** 2026-01-15

**Authors:** Joar Björk, Mikaela Friedman, Amy Nisselle, Charlotta Ingvoldstad Malmgren, Maria Johansson Soller

**Affiliations:** 1 Centre for Research Ethics & Bioethics, Uppsala University, Uppsala, Sweden; 2 Swedish National Centre for Priorities in Health, Linköping University, Linköping, Sweden; 3 Department of Research and Development, Region Kronoberg, Växjö, Sweden; 4 Department of Molecular Medicine and Surgery, Karolinska Institutet, Stockholm, Sweden; 5 Genomics in Society, Murdoch Children’s Research Institute, Melbourne, Australia; 6 Department of Paediatrics, The University of Melbourne, Melbourne, Australia

## Abstract

The mainstreaming of genomic medicine requires that non-genetics physicians take on responsibility for genomic medicine tasks. However, previous studies indicate that physicians without formal competence in genetics often lack the experience, knowledge and means to do so. This study assessed the current practice of genomic medicine among Swedish non-genetics physicians (*n* = 133) using a survey designed by McClaren et al. (McClaren et al. 2020). 67.7% of respondents had been in contact with their regional genetics services during the last year, a majority of which having been so at least monthly. Somewhat fewer, 57.1%, had ordered a gene panel, exome or whole genome during the last year. About a third reported that financing can be a barrier to ordering tests. Only 37.6% reported feeling sufficiently prepared for working with genomic medicine in general, with greatest confidence levels found for taking the medical history. Only a third of participants expected that further developments within genomic medicine will affect their own ways of working. In comparison with previous Australian and UK studies using the same survey, Swedish physicians have more contact with regional genetics services, whereas proportions ordering tests and feeling sufficiently prepared for working with genomic medicine are comparable. Interestingly, Swedish physicians seem less prepared to take on increased responsibilities for genomic medicine than their international peers. We encourage researchers in other countries to use the same survey to allow for further international comparisons to inform policy.

**Supplementary Information:**

The online version contains supplementary material available at 10.1007/s12687-026-00858-y.

## Background

Genomic medicine is evolving so rapidly that many commentators speak about a “medical genomics revolution” (Campion et al. 2019; Hyland et al. 2019). Research is increasing our knowledge about the genetic contribution to disease and carrier states at breakneck speed, and technological tools for genetic assessment continue to improve. At the same time, analytical measures which were until recently prohibitively expensive outside of research are becoming clinically available at a feasible cost (Karthikeyan et al. 2024; Monaghan et al. 2024; Reza et al. 2024). For instance, a recent Swedish micro-costing analysis showed how increased utilization can bring the price of whole-genome sequencing down considerably (Thangavelu et al. 2024). Genomic medicine is becoming embedded in routine healthcare delivery, and genomic competence is becoming more common among, for instance, oncologists and paediatricians (Johnson et al. 2017; Mateo et al. 2022). The relative lack of physicians with formal qualifications in genetics contributes to the need for a rapid diffusion of know-how from genetics centra to the clinical everyday – the so called “mainstreaming” of genomic medicine (Shkedi-Rafid et al. 2014; Hyland et al. 2019; White et al. 2020; Nisselle et al. 2021; French et al. 2023). For mainstreaming to be feasible, the knowledge of genomic medicine among physicians without formal competence in genetics (hence-forth: “non-genetics physicians”) must be sufficient and up to date, in line with aspirations of a genomic-literate workforce (Topol 2019). However, empirical results indicate that this may yet be years away. Indeed, studies from different contexts indicate that many non-genetics physicians feel they lack knowledge (Hamilton et al. 2017; Rahawi et al. 2020; Truong et al. 2021; Yu et al. 2021; Rasouly et al. 2023), have limited confidence (Johnson et al. 2017; Ha et al. 2018; de Moor et al. 2020; Maxwell et al. 2020; Whitley et al. 2020; Lopez Santibanez Jacome et al. 2022; Maher et al. 2023; Nisselle et al. 2023), and lack the competence and knowhow to interact with their regional genetics services (Carroll et al. 2016; Pasquier et al. 2022). Observational studies have revealed limitations in physicians’ knowledge and competence in discussing genetic findings (Arora et al. 2016). At the same time, studies indicate that non-genetics physicians generally have a positive attitude towards genomic medicine and find that it is relevant in their work (Hamilton et al. 2017; Spiech et al. 2020; Nisselle et al. 2021). Non-genetics physicians frequently express a need for more assistance from regional genetics services to be able to implement genomic medicine in their clinical everyday work (Johnson et al. 2017; Nisselle et al. 2021).

In the light of the above, it is imperative to investigate the possibilities and barriers regarding usage of genomic medicine among non-genetics physicians in many contexts. A well-made Australian survey study recently sought to describe the current practice of genomics, perceptions of how proximal genomic medicine is to practice, perception of preparedness and preferences for interaction with regional genetics services among Australian non-genetics physicians (Nisselle et al. 2021). The Australian survey has also been adapted to be used in the context of the National Health Services (NHS) England, again with non-genetics physicians (Bishop et al. 2023). The present study used a translated and adapted version of the Australian survey to describe the current genomic practice, knowledge and continuing education needs among Swedish non-genetics physicians. The study engendered a rich material and this article reports aspects pertaining to present and future engagement and confidence, while a separate article on education needs has already been published (Björk et al. 2025).

## Methods

### Study design

This survey study was based on a previously validated survey (McClaren et al. 2020a). The survey was translated to Swedish and slightly modified to the Swedish healthcare system, mainly regarding demographics such as the granularity of specialities and main employer. Some questions were added, which sought to gain a deeper understanding of financing of genetic testing as well as participants’ view of patients’ knowledge of genomics. The Swedish version of the survey was pilot tested by five clinical geneticists and 11 non-genetics physicians. Surface validity was assessed through cognitive interviews with four non-genetics specialists. The finalized survey had up to 174 questions, depending on branching based on question responses. Categorical, rating and open-ended questions collected data on demographics, current and preferred genomic practice, and current and preferred genomics education and training (Supplementary Materials).

### Context and participants

The survey was deployed to Swedish non-genetics physicians. All physicians in Sweden are MDs, and those with a specialisation may belong to any (or a combination of) 63 different medical specialities (Socialstyrelsen 2024). The public healthcare system in Sweden is largely tax funded. Governance is decentralized with 21 county councils having responsibility for managing their own healthcare. Many private healthcare providers have contracts with county councils to operate within the tax funded system. There are seven regional genetic services providing genetic counselling and genetic diagnostics for hematologic malignancies and different chromosomal and hereditary disorders. Whole genome sequencing is provided by three of these, but all units provide exome sequencing. Laboratory referrals for tests are sent from all levels of care, but most of them come from specialist clinics like oncology, paediatrics or gynaecology. For these patients, pre-test counselling is provided at the ordering unit, whereas post-test counselling is provided at the regional genetic service unit. Genetic counselling at the regional genetic service units is provided by specialists or trainees in clinical genetics or by genetic counsellors. There are some regional differences, for instance metabolic disorders and coagulopathies are diagnosed at specialized units outside the regional genetic services, and molecular genetic tests for solid tumours is provided by several clinical pathology units around the country. Each local care unit has a specific budget for testing with no specific adjustment for more expensive laboratory investigations, such as genetic testing. Patients only pay a fee for the medical appointment, while all further investigation including genetic testing is paid by the healthcare system. There are few alternatives for genetic testing through private sources.

Recruitment was via invitations in the Genomic Medicine Sweden Newsletter, on the organisation’s webpage and social media channels, as well as in the Swedish Medical Association’s Journal. Invitations were also sent to the specialist physicians’ associations for further dissemination. Participants were encouraged to invite further participants (snowball sampling) to reach study subjects expected to have insight and opinions on the topic. Exclusion criteria were: not being a physician; not seeing patients as part of one’s clinical practice; or having clinical genetics as primary specialty. The survey was administered online using KI-survey, a survey software developed at Karolinska Institutet. The survey was open from Nov 8th 2021 to Oct 18th 2022.

### Data cleaning and analysis

Data were downloaded, cleaned and managed in Excel (Microsoft). Analysis included descriptive statistics and standard chi-square test procedures using SPSS software (SPSS Inc). A p value of < 0.05 was considered significant. We used the definitions of engagement and confidence provided by the authors of the original survey (Nisselle et al. 2021). “Engaged” was defined as ordering a gene panel, exome or whole genome test within the last year. Being “overall confident” was defined as having an average score of “rather confident” or “very confident”, on a four-point scale used to measure confidence across four core aspects of genomic medicine (knowledge about genomics, ability to elicit information about genetic conditions as part of a family or medical history, ability to explain genomic concepts to patients and ability to make decisions based on genomic information). (This was in slight contrast to the Australian survey which used a ten-point numeric scale. Our Swedish survey simplified this to a four-point Likert-type scale with the following options: ”very unconfident” – ”rather unconfident” – ”rather confident” – ”very confident”).

**Fig. 1:**
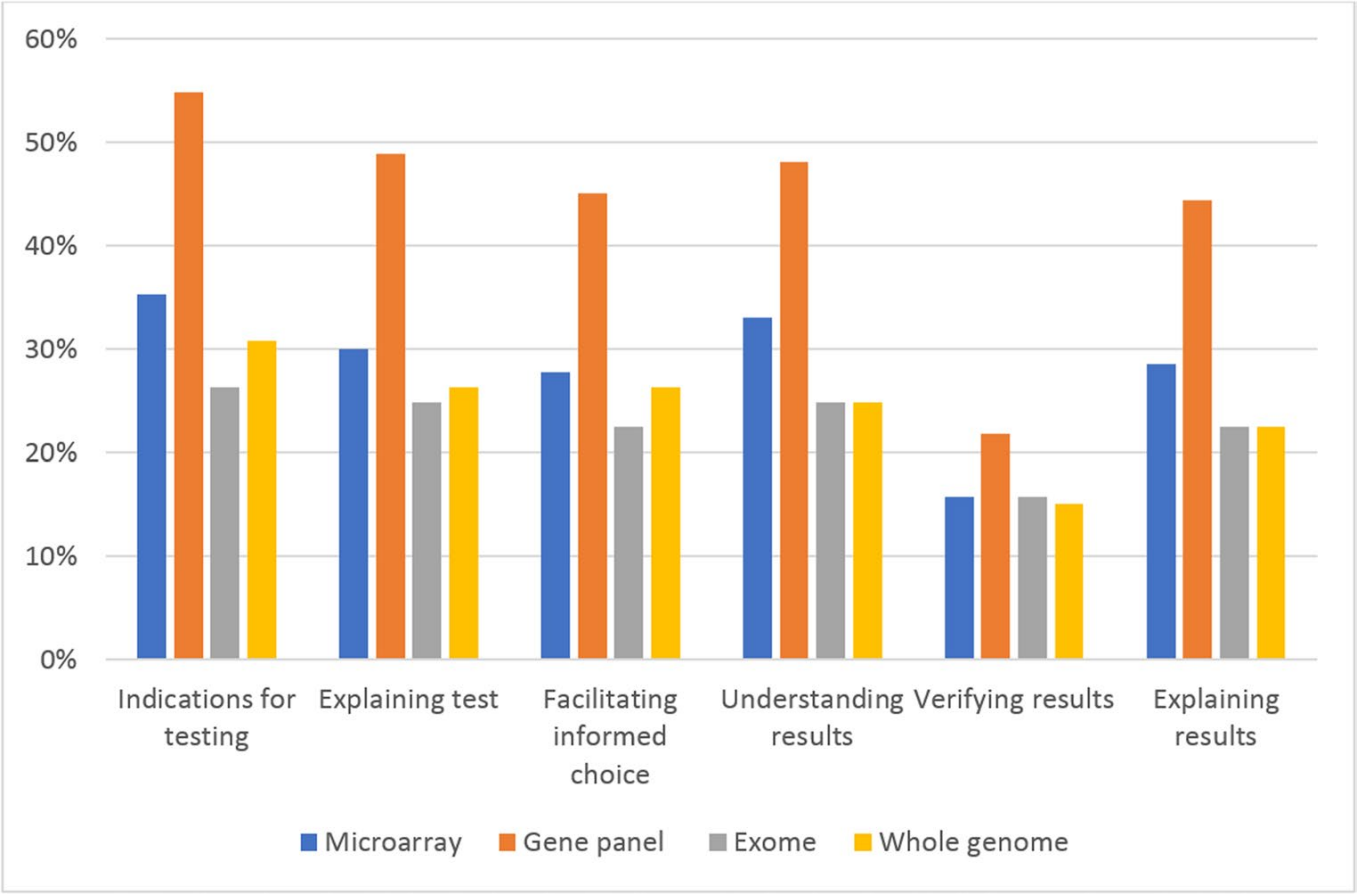
Proportions feeling rather or very confident in genomic medicine tasks; ordered by test type

We also used the 15-part categorization of tasks employed in the original survey to discuss participants current engagement in genomic medicine (see Table 2, below) as well as the original survey’s 6-part categorization of task groups to discuss confidence related to types of test (see Fig. 1, below).

### Ethics

Ethical advice was sought from the Swedish Ethical Review Authority prior to commencing the study. In their answer (reg no. 2021–04311), the authority stated that they saw no major ethical concerns. All potential participants were informed that their participation was voluntary and that no sensitive information would be collected. Data was stored within the KI Survey instrument. All answers were anonymous at the point of using the survey software, but due to the relative low numbers of some specialists in some regions of Sweden, further measures were used to ensure that no participant could be identified. The survey commenced with the question of consenting to participate in the study and give permission for data to be used for research purposes.

## Results

### Demographics

Of 179 participants who commenced the survey, five opted out of including their data for publication and 41 were excluded as they did not see patients in their practice, leaving 133 valid responses. This is thus the total number used in the Results section unless otherwise indicated due to stemming questions or missing data. Demographics are presented in Table 1. Physicians belonging to 37 specialities were represented, which is slightly more than half of all possible medical specialities in Sweden (Socialstyrelsen 2024). 41 participants had > 1 medical specialisation. Participants represented all geographical areas of Sweden, with a preponderance of participants working at university hospitals (83.5%).

**Table 1:** Demographics of the sample

GENDER	
Female	73/133 (54.9%)
Male	57/133 (42.9%)
Does not want to answer/missing	3/133 (2.3%)
AGE	
< 34	11/133 (8.3%)
35–44	35/133 (26.3%)
45–54	51/133 (38.3%)
55–64	29/133 (21.8%)
> 65	6/133 (4.5%)
CAREER STAGE	
Trainee and advanced trainee	20/133 (15.0%)
Fellow	17/133 (12.8%)
Senior consultant	96/133 (72.2%)
YEARS SINCE UNIVERSITY MEDICAL EXAM (*median*)	1–48 YEARS (*23*)
PRIMARY MEDICAL SPECIALITY	
Oncology	17/133 (12.8%)
General Paediatrics	16/133 (12%)
Obstetrics and gynaecology	15/133 (11.3%)
Internal medicine	14/133 (10.5%)
Paediatric oncology	10/133 (7.5%)
Radiology	10/133 (7.5%)
Others	51/133 (38.3%)
WORKING IN HEALTHCARE REGION	
Region Stockholm	49/133 (36.8%)
Region Uppsala	20/133 (15%)
Region Skåne	20/133 (15%)
Västra Götalandsregionen	18/133 (13.5%)
Region Östergötland	7/133 (5.3%)
Västra Västerbotten	6/133 (4.5%)
Region Örebro län	6/133 (4.5%)
Others	7/133 (5.3%)
MAIN EMPLOYER	
University Hospital	111/133 (83.5%)
Non-university Hospital	9/133 (6.8%)
University or other research institution	5/133 (3.8%)
Other	8/133 (6%)

#### Current practice of genomic medicine

A large proportion of participants in this study were involved with genomic medicine in some form. In regards to scientific engagement and teaching, 42.9% of participants (57/133) had been involved in genomic medicine research within the last five years, and 17.3% (23/133) had been involved in genomic medicine teaching during the last year. In terms of clinical engagement, 67.7% (90/133) had been in contact with their regional genetics services during the last year. Of those who had been in contact, 56.7% (51/90) had been in contact at least monthly. The two most common reasons for contact was to order a test 63.3% (57/90), or to discuss whether a test should be ordered and if so which test (47.8%; 42/90). Of those who stated they had *not* been in contact with their regional genetics services (*n* = 40), the most common reason provided (82.5%; 33/40) was that they had not had the need to do so, with 17.5% (7/40) not knowing how to contact the services, 12.5% (5/40) stating that they can manage genomic patients without help and 5.0% (2/40) stating they have no access to regional genetics services. 15.0% (6/40) stated there were “other reasons”. (More than one answer could be given).

As for participants’ current engagement in *the clinical practice* of genomic medicine, 66.9% (89/133) were involved in a task from the pre-test, test or post-test phase (for a list of tasks in the three phases see Table 2). 57.9% (77/133) were currently involved in *pre-test* work, most commonly taking a patient’s family history (54.9%; 73/133) and identifying whether a patient is suitable for genomic testing (47.4%; 63/133). The proportion who were “engaged”, i.e., had ordered a gene panel, exome or whole genome within the last year, was 57.1% (76/133). Only two participants had *only* ordered the microarray test, and hence the proportion who had ordered any form test within the last year was 58.6% (78/133).

**Table 2:** Tasks in the clinical practice of genomic medicine (adapted from McClaren et al. 2020)

Pre-test phase
Taking patient’s family history
Identifying whether patient is suitable for genomic testing
Counselling to help patient make informed decision about testing
Ordering a genomic test for a patient
**Test phase**
Attending multidisciplinary team (MDT) meeting to discuss indications for testing
Assisting the lab to narrow down genes of interest in the particular case
Providing phenotypic information about patient to prioritise variant analysis
Searching information on pathogenicity in literature and databases
Attending MDT to discuss *variant prioritisation*, interpretation and *classification*
**Post-test phase**
Providing test results to patients/families
Providing *genetic counselling* to patients/families
Organizing/referring for further testing of family members
Ongoing management of the patient, e.g., clarify recurrence risk and discuss reproductive planning options
Assessing patient’s/family’s understanding and answering additional questions

The test ordered by most participants was the gene panel (54.1%, 72/133) followed by microarray (29.3%, 39/133), whole genome (28.6%, 38/133) and whole exome (18.8%, 25/133). Most tests were ordered independently without support from regional genetics services apart from whole exome tests, where 60.0% (15/25) of ordering physicians had done this with assistance from their regional genetics service. 17.3% (23/133) had *only* ordered a gene panel, whereas only 3 and 2 participants, respectively, had ordered the whole genome test or microarray and no other form of genomic test. No participant had ordered merely an exome test. 12.8% (17/133) had ordered *all* four kinds of genomic tests and 12.0% (16/133) were “frequent users”, having ordered at least one form of test daily or weekly.

Financing can be a barrier to ordering genomic tests. 29.3% (39/133) reported that cost was a significant hindrance to ordering genomic tests. Of these, however, 59.0% (23/39) reported that some tests can still be ordered after, for instance, team discussions. Free text answers indicated that other barriers include not knowing the indication, not knowing how to order tests and not knowing how to contact regional genetics services.

In comparison with tasks from the pre-test phase, slightly fewer participants (48.1%; 64/133) were involved in any task from the test phase (Table 2). Participating in a multidisciplinary team meeting and providing phenotypic information were the most common activities, which 33.8% (45/133) and 32.3% (43/133) of participants were involved in, respectively. Fewer still (40.6%, 54/133), were involved in any task from the post-test phase, with providing test results to patients/families being the most common (34.6%, 46/133).

#### Confidence

Despite a large proportion being “engaged” with genomic medicine, only 37.6% (50/133) reported feeling sufficiently prepared for working with genomic medicine in general. Similarly, only 54.9% (73/133) of participants were “overall confident” (see Methods) in practising genomic medicine. The aspect in which most participants were rather or very confident was their ability to elicit information about genetic conditions as part of taking medical history (55.6%; 74/133). Somewhat lower proportions were rather or very confident in their knowledge about genomics (50.4%; 67/133), ability to explain genomic concepts to patients (47.4%; 63/133) and ability to make decisions for patient management based on genomic information (45.1%; 60/133).

When confidence was assessed per type of test, the gene panel was the test that most participants felt confident with (48.9%; 65/133 feeling rather or very confident with at least half of the tasks related to this type of test, and 18% (24/133) feeling rather or very confident in *all* tasks related to this type of test), whereas exome was the test that *least* participants felt confident with. Indeed, only 26.3% (35/133) felt rather or very confident in at least half of the tasks related to this type of test. Figure 1 shows confidence levels by type of test.

#### The future of genomic medicine

A significant proportion of participants believed that genomics will become more relevant in the future, in a number of ways. 51.1% (68/133) believed that they themselves will order more genomic tests, and almost as many that they will order more pharmacogenomic tests and/or contact genetics services more (50.4%; 67/133 for both answers). In addition, they believed that patients will ask about having tests ordered for them or have questions about direct-to-consumer tests (52.6%; 70/133 and 34.6%; 46/133, respectively). Free text comments about why they believe that the field will change reflected an expectation that new therapies will be discovered, that research in the area is “very fast”, that treatments will become individualised, and that patients will demand it. Indeed, 23.3% (31/133) of all participants expected to take on some genomic medicine task related to the pre-test phase that *they do not currently perform* (for tasks within the different phases, see Table 2). The most common such task to be expected was ordering a genomic test they did not currently order (18%, 24/133) Even more participants expected to take on some task related to the test phase (30.8%, 41/133), most commonly to take part in multidisciplinary team meetings (27.8%, 37/133). Finally, 21.8% (29/133) expected to take on some task related to the post-test phase, most commonly providing test results to patients/families (12.8%, 17/133).

At the same time, most participants did not expect that genomic medicine will affect their own work. Only 33.8% (45/133) indicated that genomic medicine will affect their own way of working, and a mere 21.1% (28/133) that it will affect their own workload.

#### Between-group comparisons

Chi-square tests were performed to examine possible between-group differences. Paediatricians were more likely to be “engaged” and “overall confident” (*X*^*2*^ = 26.6; p = < 0.00001; *X*^*2*^ = 5.79; *p* =.016, respectively), and more likely to believe that physicians will order more genomic tests within the coming years than were non-paediatricians (*X*^*2*^ = 4.82; *p* =.028). Unsurprisingly, participants who were involved in genomics teaching were more often involved in genomics research, were more often “engaged” and “overall confident” and were more often “frequent users” than non-teachers (*X*^*2*^ = 21.89; p = < 0.00001; *X*^*2*^ = 13.15; *p* =.00029; *X*^*2*^ = 10.48; *p* =.0012; *X*^*2*^ = 34.98; p = < 0.00001; respectively). Those who were “engaged” were more commonly involved in genomics research than non-engaged participants (*X*^*2*^ = 23.37; p = < 0.00001). “Frequent users” were more often “engaged” and “overall confident” than other participants (*X*^*2*^ = 13.64; *p* =.00022 and *X*^*2*^ = 7.81; *p* =.0052). In contrast, gender or years working as a physician did not appear to affect being “engaged”, “overall confident” or involved in teaching. None of the examined subgroup comparisons showed any correlations with believing that one’s own workload in genomic medicine will increase.

## Discussion

This survey of Swedish physicians not specialised in genetics shows that a large percentage are already using genomic medicine, while at the same time many report feeling unprepared for, and lacking confidence in, using genomic medicine. We will now proceed to discuss these results and contextualise them with relevant international studies. The most important comparators are the studies deploying the same survey, i.e. the Australian study by Nisselle and colleagues (reported in Nisselle et al. 2021; Nisselle et al. 2023); these will henceforth be referred to as “the Australian study”, but referenced by individual article) and “the UK study” by Bishop and colleagues (Bishop et al. 2023).

The sample size in our study was comparable, in absolute figures, to the UK study but smaller than the Australian study. As Sweden has fewer physicians than either of these countries, our study sample represents a larger portion of the physician population. The age and qualification demographics are comparable across the three studies. Women are somewhat more frequent in the Swedish sample than in the Australian and UK studies, reflecting national differences in the physician workforce (Socialstyrelsen 2024). In terms of current involvement with genomic medicine, it is notable that participants in the Swedish sample were somewhat more involved in genomic research than in the Australian sample (42.9% versus 24.7%). This may be related to the rapid spread of genomic medicine, as some years have passed between the studies, the fact that a large proportion of participants in the Swedish sample were employed at university hospitals (83.5%), or an artefact of the snowball sampling procedure. Nonetheless, the fact that we report on a strongly research-oriented group of participants must be kept in mind when comparing other results between Sweden, Australia and the UK.

Proportions of participants who had been in contact with regional genetics services in the past year were higher in Sweden than in Australia and the UK (67.7% as compared to 52.5% and 54.4% respectively). The proportions who had ordered any form of genetic or genomic test were also somewhat higher in Sweden than in the UK (58.6% compared to 54.2% in the UK, data for Australia lacking). Proportions being “engaged” were comparable between Sweden and Australia (57.1% and 53.9% respectively, data for UK lacking). Interestingly, test popularity differed widely with Swedish non-genetics physicians reporting ordering considerably more gene panels than any other form of test, whereas their international peers ordered about the same proportions of gene panels and microarrays. This is likely a combination of differing local traditions (Fioretos et al. 2022) and the fact that there is a time gap between the Australian survey and the Swedish survey. In all three studies, exome or whole genome testing were the most infrequently ordered tests, which has also been shown in other studies (Maher et al. 2023). That non-genetics physicians are currently most involved in pre-test aspects of genomic medicine is consistent with the Australian study.

That large proportions of the non-genetics physicians feel insufficiently prepared to practise genomic medicine is well known from previous studies (e.g. de Moor et al. 2020; Lopez Santibanez Jacome et al. 2022; Rahawi et al. 2020; Rasouly et al. 2023; Truong et al. 2021; Yu et al. 2021). In the Swedish sample, 37.6% felt sufficiently prepared, compared to 24.6% in the Australian sample and 45.8% in the UK sample. The larger proportion in the UK may reflect the country’s forward leaning approach to genomics education (Karthikeyan et al. 2024). “Overall confidence” was 54.9% in Sweden as compared to 42% in Australia. Other studies from more specialised cohorts have evidenced higher levels of confidence (Ha et al. 2018; de Moor et al. 2020). The feeling of being unprepared and lacking confidence is cause for concern in several ways. It is easy to see how this can hamper the necessary “mainstreaming” of genomic medicine, as previous studies show that preparedness and confidence influence behaviour in genomic medicine (Schaibley et al. 2022; Nisselle et al. 2023). It is also disconcerting at the very human level, as previous qualitative research indicates that limited knowledge and confidence in genomic medicine makes non-genetics physicians feel unhappy (Carroll et al. 2016; Pasquier et al. 2022).

In line with the Australian, UK and other studies, confidence varies by task and type of test, and this study affirms previous indications that those who most frequently order tests are more confident (de Moor et al. 2020; Hall et al. 2021). In terms of different types of tasks, Swedish participants were generally less confident than their Australian and UK counterparts, but the pattern with highest confidence in eliciting information as part of taking a medical history and the lowest confidence in the ability to make medical decisions based on genomic information is consistent across the three study sites. The relatively high level of competence in taking a medical history may be related to the fact that this, in part, relies on generalist communication competencies, which many physicians may feel comfortable with due to training and experience (Chambers et al. 2015). As for the lower reported confidence in individual skills in Sweden, it should be kept in mind that lower confidence does not necessarily translate to lower competence (Ehrlinger et al. 2008). Answering patterns may also be influenced by, for instance, social desirability bias (Clanton et al. 2014). Swedish culture is generally known as one which discourages boasting (Scott 2022). In spite of the tendency for men to rate their own competencies higher than females (Vajapey et al. 2020), there was no statistically significant gender differences in this material in any of the knowledge or confidence measures. Interestingly, there was also no correlation between years of practise and knowledge or confidence, in contrast to some previous studies (Ha et al. 2018). However, there was a correlation between having a paediatric speciality and feeling better prepared to work with genomic medicine. This pattern is consistent with the Australian study and others (Rasouly et al. 2023), whereas the UK study did not report on the matter. Several authors have noted that this may have to do with the greater relevance of genetics in paediatrics currently (Rimoin and Hirschhorn 2004; Eichinger et al. 2021; Hodder et al. 2024).

A large proportion of participants in this study believed, in line with the Australian and UK studies and many others (Rahawi et al. 2020; Yu et al. 2021; Schaibley et al. 2022; French et al. 2023), that genomic medicine will become even more clinically relevant within the near future. Many Swedish non-genetics physicians expect to take on more genomic medicine tasks in the future. Interestingly, they are mainly expecting to perform more tasks from the pre-test and test phases, although they may well have to take on larger post-test responsibilities as well, at least when it comes to providing normal results. Indeed, if more pre-test work is undertaken – by anybody –more post-test work will also need to be done. It is worrying that many Swedish non-geneticists seem to expect their workload will remain unchanged despite increased progress of genomic medicine. Even though pre-test information may be standardized to save time, post-test information will often be complex and hard to standardize. It is not realistic to assume that Swedish clinical geneticists and genetic counsellors will be able to carry the full burden, as their number is not expected to grow substantially in the years ahead. Indeed, the Australian and UK answering patterns suggest non-genetics physicians in these countries are more prepared to take on an increased work load in genomic medicine. In general, applauding all positive aspects while negating the costs (in any sense) of genomic medicine may contribute to a “genohype” (Stark et al. 2019). In this spirit, free text answers in this survey indicated non-genetics physicians feel they and their patients do not receive adequate support when it comes to genomic medicine. One possible way forward may be to further the integration of genetic counselling in genomic practice, for the benefit of both physicians and patients (Paneque et al. 2017).

### Methodological reflections

A strength of this study is the relatively large number of participants from a wide range of medical specialities. The fact that a majority were “engaged” indicates that the sampling yielded a study population which is relevant to study when it comes to the mainstreaming of genomic medicine. At the same time, answering patterns may be less generalisable to non-genetics physicians whose proximity to genomic medicine is lower, such as GPs or physicians not working at university hospitals. Another strength was the use of a theory- and empirically-informed, previously validated survey, which allows comparison between varied locations and contexts, and benchmarking across time. The fact that the survey was rather long could have influenced participation and perhaps answering patterns (Bishop et al. 2023). At the same time, the comprehensive survey gives very good insight into the studied phenomenon. We acknowledge that questions pertaining to “multidisciplinary team meetings” are ambiguous as we did not specify whether such meetings should include a genetics specialist or not.

## Conclusion

This survey study indicates that Swedish non-geneticists are already to a great extent engaged with genomic medicine, but have limited confidence in their knowledge and skills in the area. Results are largely comparable to studies from Australia and the UK. Swedish participants contact regional genetics services and order genomic tests at least as much as their international peers. Taken as a whole, our results suggest there is a need to improve Swedish practices regarding genomic medicine when it comes to education, support, referral patterns and contact pathways. The interplay between non-genetics physicians and their counterparts in regional genetics services may need to change to harvest the potential benefits of genomic medicine. As part of the urgently-needed discussion about mainstreaming of genomic medicine in Sweden and internationally, we encourage researchers in other countries to use the same survey to describe the terrain in their contexts and allow international comparisons to inform policy.

## Supplementary Information

Below is the link to the electronic supplementary material.

Supplementary Material 1

## Data Availability

“The Swedish version of the survey can be found in the supplementary information files”.
